# Modifiable patient-related barriers and their association with breast cancer detection practices among Ugandan women without a diagnosis of breast cancer

**DOI:** 10.1371/journal.pone.0217938

**Published:** 2019-06-20

**Authors:** Jake W. Sharp, Daniel S. Hippe, Gertrude Nakigudde, Benjamin O. Anderson, Zeridah Muyinda, Yamile Molina, John R. Scheel

**Affiliations:** 1 Department of Radiology, University of Washington, Seattle, Washington, United States of America; 2 Uganda Women’s Cancer Support Organisation (UWOCASO), Kampala, Uganda; 3 Division of Public Health Sciences, Fred Hutchinson Cancer Research Center, Seattle, Washington, United States of America; 4 Department of Surgery, University of Washington, Seattle, Washington, United States of America; 5 Department of Global Health, University of Washington, Seattle, Washington, United States of America; 6 Department of Radiology, Mulago Hospital, Kampala, Uganda; 7 Community Health Sciences Division, School of Public Health, University of Illinois at Chicago, Chicago, Illinois, United States of America; University of Chicago, UNITED STATES

## Abstract

Most women with breast cancer in sub-Saharan Africa (SSA) are diagnosed with late-staged disease. The current study assesses patient-related barriers among women from a general SSA population to better understand how patient-related barriers contribute to diagnostic delays. Using convenience-based sampling, 401 Ugandan women without breast cancer were surveyed to determine how prior participation in cancer detection practices correlate with patient-related barriers to prompt diagnosis. In a predominantly poor (76%) and rural population (75%), the median age of the participants was 38. Of the women surveyed, 155 (46%) had prior exposure to breast cancer education, 92 (27%) performed breast self-examination (BSE) and 68 (20%) had undergone a recent clinical breast examination (CBE), breast ultrasound or breast biopsy. The most commonly identified barriers to prompt diagnosis were knowledge deficits regarding early diagnosis (79%), economic barriers to accessing care (68%), fear (37%) and poor social support (24%). However, only women who reported knowledge deficits—a modifiable barrier—were less likely to participate in cancer detection practices (p<0.05). Women in urban and rural areas were similarly likely to report economic barriers, knowledge deficits and/or poor social support, but rural women were less likely than urban women to have received breast cancer education and/or perform BSE (p<0.001). Women who have had prior breast cancer education (p<0.001) and/or who perform BSE (p = 0.02) were more likely to know where she can go to receive a diagnostic breast evaluation. These findings suggest that SSA countries developing early breast cancer detection programs should specifically address modifiable knowledge deficits among women less likely to achieve a diagnostic work-up to reduce diagnostic delays and improve breast cancer outcomes.

## Introduction

**Fig 1:**
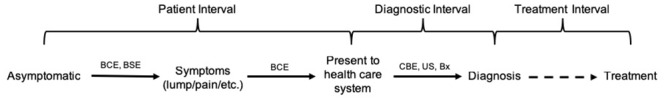
A Diagram Illustrating the diagnostic pathway. Education and breast self-exam lead to recognition of symptoms and breast cancer education facilitates women presenting to the health care system for evaluations (Patient interval). Delays in the patient interval can be caused by patient factors and health system factors, such as location of health facilities. The diagnostic interval extends from a woman presenting to the health system until she achieves a diagnosis, and this interval also includes both patient and health system factors. Treatment interval encompasses the time between a woman achieving a diagnosis and the initiation of treatment. BCE: breast cancer education; BSE: breast self-exam; CBE: clinical breast exam; US: breast ultrasound; Bx: biopsy.

When patients present with late stage breast cancer, the treatment is more resource-intensive and less likely to result in a cure. In Uganda, up to 89% of women with breast cancer present for treatment at a late stage as the result of a delay in their path to diagnosis.[1–3] The patient pathway for early diagnosis (Fig 1) consists of two phases including the patient interval (time between the development of breast symptoms and presentation to a health center) and the diagnostic interval (time between presentation to a health center and completion of a diagnostic work-up)[4], with mean patient and diagnostic intervals of 29 months[5] and >6 months, respectively.[6] Each phase is associated with specific breast cancer detection practices that in unscreened populations are associated with increased rates of successful early diagnosis. However, while breast self-examination (BSE) is associated in the patient interval and clinical breast examination (CBE), breast ultrasound and biopsy are important in the diagnostic interval, breast cancer education is essential throughout the patient pathway for early diagnosis, particularly in countries where the referral pathways are not established, such as in sub-Saharan Africa. Given the strong association between delays in the patient pathway for early diagnosis and poor outcomes,[1, 3] shortening the patient and/or diagnostic intervals has potential to improve breast cancer survival.

Prior studies on barriers that Ugandan women encounter in their path to diagnosis have only included women with breast cancer who achieved a diagnostic work-up and received cancer treatment,[5, 7] which account for only 14% of women with breast cancer.[8] Additionally, many of these studies have predominantly selected women from the major urban center of Kampala to the exclusion of rural populations- the majority of Ugandan women. Barriers preventing the majority of women from making it to treatment are unknown. Literature from the U.S. and Europe is not applicable since most breast cancer is detected early through population screening, high awareness, and improved access to healthcare—a logistic impossibility in Uganda where only BSE and CBE are available as detection methods.[9, 10] Furthermore, socially determined factors (e.g., economic barriers to accessing care) in the U.S. and Europe are particularly important to early diagnosis, yet may be less important in SSA where economic barriers are ubiquitous. The effect of barriers on participating in breast cancer detection practices is rarely assessed in SSA as most research is qualitative or descriptive. Assessing barriers women without breast cancer encounter, as it relates to participating in breast cancer detection practices, will help program planners understand the barriers women with breast cancer encounter who are less likely to complete the path to diagnosis- the most common outcome for women with breast cancer.

While breast self-examinations (BSE) and clinical breast examinations (CBE) are no longer recommended for screening in high income countries with access to screening mammography, both are essential and obligatory precursors for early diagnosis in low resource countries.[9] Both also represent key indicators of “awareness” and interaction with the health system, respectively. In addition, CBE represents an important time for clinicians to educate patients about breast health. Both the American Cancer Society and NCCN recommend “awareness”, and both BSE and CBE are recommended in the breast cancer detection guidelines for Uganda. [10]

In accordance with recommendations by the Breast Health Global Initiative (BHGI) to target social-cultural barriers that contribute to poor breast cancer outcomes,[11] our group surveyed Ugandan women without breast cancer to assess potential barriers along the path to diagnosis. We previously reported lower participation in breast cancer detection practices in women without breast cancer, compared to prior research done in women with breast cancer who accessed treatment,[12] and their participation was further reduced with increased family obligation stressors (i.e., the perceived needs of the family supersede self-care).[13] Similarly, most women believed in cultural explanations for breast cancer (e.g. that carrying money in bras causes breast cancer) rather than scientific causes (e.g. older age, genetics). Also, while many women believed that early detection would result in a cure, most believed by the time they self-detected symptoms it was too late.[14] These results, obtained in women without breast cancer, suggested multiple modifiable factors that could affect women’s participation in breast cancer detection practices. Simultaneously, these results seem to contradict the multiple barriers, not modifiable in the near term (socially determined barriers), Ugandan breast cancer survivors and others provided in prior studies (e.g., economic, social support) as possible reasons most women delay diagnosis or do not successfully complete the path to diagnosis.

In the current study, we assess prevalence of patient-related barriers and their associations with breast cancer detection practices in women without breast cancer as indicators of successful progress towards diagnosis.

## Methods

### Ethics

The survey was reviewed and approved by Ugandan (Makerere University College of Health Sciences Research and Ethics Committee) and US (University of Washington Human Subject Division) institutional review boards and the Uganda National Council for Science and Technology (UNCST). Written and/or documenting consent was waived because this was a minimal risk study and it would have been the only link between the participant and the study introducing a small potential risk of loss of confidentiality. The UWOCASO volunteer solicited verbal consent to each participant individually.

### Participants and setting

The protocol was described previously.[12] Briefly, we partnered with the Ugandan Women’s Cancer Support Organization (UWOCASO), a group of breast cancer survivors who promote community breast cancer awareness, fundraise to support breast cancer related activities, and act as patient navigators. UWOCASO helped develop and test the Attitudes on Breast Cancer Surveillance and Knowledge (ASK) survey, then recruited participants from a variety of settings (e.g., market places, health fairs) using a convenience-based sampling approach between January and July 2014.

We included women age 25 years and older with no personal history of breast cancer. Trained UWOCASO members interviewed eligible women individually in a semiprivate area. There were 401 participants surveyed: 100 women from the urban capital of Kampala and 301 from rural villages in south central Uganda (Kakuuto and Kooki counties). The ratio of rural:urban women was selected to approximate the proportion of rural women in Uganda (80%). The locations for recruitment in both the urban and rural settings were intentionally selected to increase the probability of sampling poor women and approximate the 70% of Ugandans who are poor or are vulnerable to poverty.[15] These populations of women are more vulnerable to diagnostic delays and poorer outcomes. [16, 17] Participating women received ~5 US dollars for travel and their time in accordance with local recommendations.

### Measures

The development and testing of the ASK survey have been described previously.[12] In summary, standard methods of cross-cultural adaptation and development of surveys were used[18–27] along with previously published data[16, 28] and focus groups guided by trained facilitators to identify key constructs related to barriers in breast cancer detection practices. A panel of experts reviewed the barriers these methods identified and recommended additional content for survey items, as needed. The final ASK survey was translated from English (primary language) to Luganda (common local language).

For breast cancer detection practices, women were asked if they had received prior education about breast cancer (yes, no); if they performed a BSE and, if so, the frequency (recoded as ever, never); if they received a CBE in the past year (recent CBE: yes, no); if they ever received a breast ultrasound (yes, no); and if they ever received a breast biopsy (yes, no). Participants were also asked whether they agreed, disagreed, or were unsure about statements related to barriers against participating in breast cancer detection (worded such that agreement was the “incorrect” or undesirable answer). These statements related to barriers were clustered into barrier types (“Economic barriers to accessing care,” “Knowledge deficits,” “Poor social support,” and “Fear”) by author consensus (JWS, DH, YM, and JRS).

### Data analysis

The Collaborative Data Services at the Fred Hutchinson Cancer Research Center entered the survey data into the DatStat Illume software package (Seattle, WA). A barrier was considered present if the respondent answered “agree” or “unsure” to the corresponding barrier question (undesirable response) and absent if she answered “disagree.” Answers were combined against “disagree” (desirable response). The presence of each barrier and barrier type were compared between groups using Fisher’s exact test. Binomial regression was used to adjust group comparisons of outcomes for urban/rural setting and age. Differences were presented as probability differences (PDs), with standard errors calculated using robust sandwich estimator. The number of barrier types was compared between groups using the Wilcoxon rank-sum test. All statistical calculations were conducted with the statistical computing language R (version 3.1.1; R Foundation for Statistical Computing, Vienna, Austria). Statistical significance was defined at a p-value of 0.05 or less without adjustment for the number of comparisons.

## Results

### Population characteristics and participation in different breast cancer detection practices

The sociodemographic characteristics of the included and excluded respondents are summarized in Table 1. Of the 401 surveyed, 341 (85%) women were included in the analysis after exclusion for incomplete survey responses to barrier-related questions. There were no statistically significant differences in the assessed characteristics between the women included and excluded.

**Table 1:** Population characteristics and experiences with breast cancer detection practices.

	Samples N = 401*			Location N = 341*		
Variable	Included N = 341	Excluded N = 60	P-value	Urban N = 83	Rural N = 258	P-value
Age			0.58			0.60
25–39	182 (53.4)	33 (55.0)		41 (49.4)	141 (54.7)	
40–49	89 (26.1)	18 (30.0)		25 (30.1)	64 (24.8)	
50–74	70 (20.5)	9 (15.0)		17 (20.5)	53 (20.5)	
Ethnicity†			>0.99			**<0.001**
Bantu	301 (89.3)	54 (90.0)		63 (75.9)	238 (93.7)	
Other	36 (10.7)	6 (10.0)		20 (24.1)	16 (6.3)	
Religion†			0.67			**<0.001**
Catholic	211 (62.1)	37 (61.7)		29 (34.9)	182 (70.8)	
Protestant	90 (26.5)	14 (23.3)		41 (49.4)	49 (19.1)	
Muslim	39 (11.5)	9 (15.0)		13 (15.7)	26 (10.1)	
Education†			>0.99			0.13
≤Primary (P1-P7)	236 (69.4)	42 (70.0)		52 (62.7)	184 (71.6)	
>Primary (>P7)	104 (30.6)	18 (30.0)		31 (37.3)	73 (28.4)	
Geographic Location			0.52			
Urban	83 (24.3)	17 (28.3)				
Rural	258 (75.7)	43 (71.7)				
Employed full time/student†			0.096			0.33
Yes	235 (70.4)	35 (59.3)		52 (65.8)	183 (71.8)	
No	99 (29.6)	24 (40.7)		27 (34.2)	72 (28.2)	
Married/living with significant partner†			0.39			0.29
Yes	207 (61.6)	40 (67.8)		45 (56.2)	162 (63.3)	
No	129 (38.4)	19 (32.2)		35 (43.8)	94 (36.7)	
Household income†			0.10			0.059
0–100,000 shillings	56 (22.2)	15 (33.3)		7 (11.7)	49 (25.5)	
100,001–500,000 shillings	69 (27.4)	10 (22.2)		15 (25.0)	54 (28.1)	
500,001–1,000,000 shillings	66 (26.2)	15 (33.3)		18 (30.0)	48 (25.0)	
>1,000,000 shillings	61 (24.2)	5 (11.1)		20 (33.3)	41 (21.4)	
Received any breast cancer education			0.11			**<0.001**
Yes	155 (45.5)	26 (59.1)		57 (68.7)	98 (38.0)	
No	186 (54.5)	18 (40.9)		26 (31.3)	160 (62.0)	
Performs BSE			0.73			**<0.001**
Yes	92 (27.0)	11 (23.9)		39 (47.0)	53 (20.5)	
No	249 (73.0)	35 (76.1)		44 (53.0)	205 (79.5)	
CBE within the last year			>0.99			**<0.001**
Yes	53 (15.5)	8 (14.5)		30 (36.1)	23 (8.9)	
No	288 (84.5)	47 (85.5)		53 (63.9)	235 (91.1)	
Prior breast ultrasound			0.72			>0.99
Yes	16 (4.7)	3 (6.0)		4 (4.8)	12 (4.7)	
No	325 (95.3)	47 (94.0)		79 (95.2)	246 (95.3)	
Prior breast biopsy			0.43			**0.006**
Yes	11 (3.2)	3 (5.3)		7 (8.4)	4 (1.6)	
No	330 (96.8)	54 (94.7)		76 (91.6)	254 (98.4)	

BSE = breast-self exam; CBE = clinical breast exam;

*Values are no. (%) or mean ± SD;

^†^Respondents with missing values were excluded from the corresponding summary; in the included group, the following variables had missing values: ethnicity (n = 4), religion (n = 1), education (n = 1), employment status (n = 7), marital status (n = 5), and income (n = 89)

Table 1 also characterizes the study population’s experiences with different breast cancer detection practices as a group and by geographic location (urban versus rural). In general, the proportion of women not participating in breast cancer detection practices increased as the method became more invasive: had no prior breast cancer education (55%), do not perform a BSE (73%), had not received a recent CBE (85%), had never had an US (95%) and never had a biopsy (97%). We found rural women, compared to urban women, were significantly less likely to have participated in most breast cancer detection practices: breast cancer education (p<0.001), BSE (p<0.001), recent CBE (p<0.001), and biopsy (p = 0.006).

### Frequency of type and number of barriers to breast cancer detection in urban and rural women

Table 2 shows the frequency of women reporting barriers related to knowledge deficits, economic barriers to accessing care, poor social support, and fear. The most commonly held barriers were related to knowledge deficits (79%), followed by economic barriers to accessing care (68%), fear (37%), and poor social support (24%). “Heard bad things about healthcare” was included as a reason why one might not seek healthcare. However, only 9.6% of women included this response and no one reported this as a “most important” reason for not seeking healthcare. Of the participants, 8% of women reported no barriers, 21% reported a single barrier, 38% two barriers, 21% three barriers, and 13% four barriers.

**Table 2:** Patient-related barriers to breast cancer early detection.

Barriers	Responses	Agree/Unsure†	Disagree†
Economic barriers			
The cost of getting a breast exam keeps me from getting one.	341	233 (68.3)	108 (31.7)
Poor social support	341	82 (24.0)	259 (76.0)
My partner does not want me to get a breast exam.	340	38 (11.2)	302 (88.8)
Not being able to take time off from work keeps me from getting a breast exam	341	58 (17.0)	283 (83.0)
Knowledge deficits	341	270 (79.2)	71 (20.8)
A breast exam is not recommended for women my age.	338	68 (20.1)	270 (79.9)
I only need a breast exam if I have breast problem.	341	157 (46.1)	184 (54.0)
I don’t know where I should go if I want to get a breast exam.	340	172 (50.6)	168 (49.4)
I don’t need a breast exam from a doctor because I examine my own breasts.	336	33 (9.8)	303 (90.2)
I do not need a breast exam because I feel fine.	337	46 (13.7)	291 (86.4)
Fear/psychological	341	127 (37.3)	214 (62.8)
Feeling embarrassed keeps me from getting a breast exam.	339	24 (7.1)	315 (92.9)
The pain of a breast exam is what keeps me from getting one.	340	55 (16.2)	285 (83.8)
I do not get a breast exam because I am afraid they will find cancer.	334	33 (9.9)	301 (90.1)
I am afraid of getting an ultrasound because I may have cancer.	340	38 (11.2)	302 (88.8)
A breast ultrasound would not give me peace of mind.	338	51 (15.1)	287 (84.9)

BSE = breast self-exam; CBE = clinical breast exam; US = breast ultrasound;

^†^Values are no. (%) or mean ± SD;

Urban women were significantly more likely to report barriers related to fear (52% v. 33%; p = 0.003). Otherwise, urban and rural women reported no significant differences in barrier types (S1 Table). There was also no significant difference in the number of reported barriers between urban and rural women.

### Associations among barrier-type and participation in different breast cancer detection practices

Because socially determined barriers, such as economic barriers to access and poor social support, would have different implications to early breast cancer detection than modifiable barriers such as knowledge deficits and fear, we next tested for associations between barrier types and participation in breast cancer detection practices (Table 3). Only knowledge deficits were significantly associated with decreased participation in breast cancer detection practices in the patient interval (breast cancer education, p = 0.005 and BSE, p = 0.002), and trended toward significance in breast cancer detection practices in the diagnostic interval (CBE, breast ultrasound, or breast biopsy, p = 0.066). Socially determined barriers, such as economic barriers to accessing care and poor social support, and a potentially modifiable barrier, fear, were not significantly associated with participation in breast cancer detection practices. The number of barrier types were also not significantly associated with participating in breast cancer detection practices. Adjusting for potential differences among women living in a rural versus urban setting and age had little impact on associations with participation in breast cancer detection practices (S2 Table).

**Table 3:** Associations between patient-related barriers and participation in breast cancer detection practices.

	Breast Cancer Education†			Regular BSE†			Recent CBE or Prior Breast US or Biopsy†		
Barriers	Yes (N = 155)	No (N = 186)	P-value‡	Yes (N = 92)	No (N = 249)	P-value‡	Yes (N = 68)	No (N = 273)	P-value‡
Economic barriers	99 (63.9)	134 (72.0)	0.13	59 (64.1)	174 (69.9)	0.36	46 (67.6)	187 (68.5)	0.89
Poor social support	38 (24.5)	44 (23.7)	0.90	18 (19.6)	64 (25.7)	0.26	20 (29.4)	62 (22.7)	0.27
Knowledge deficits	112 (72.3)	158 (84.9)	**0.005**	62 (67.4)	208 (83.5)	**0.002**	48 (70.6)	222 (81.3)	0.066
Fear	62 (40.0)	65 (34.9)	0.37	36 (39.1)	91 (36.5)	0.71	30 (44.1)	97 (35.5)	0.21
Number of barrier types	2.0 ± 1.1	2.2 ± 1.1	0.26	1.9 ± 1.2	2.2 ± 1.1	0.092	2.1 ± 1.2	2.1 ± 1.1	0.72

BSE = breast self-exam; CBE = clinical breast exam; US = breast ultrasound;

^†^Values are no. (%) or mean ± SD;

^‡^Fisher’s exact test (presence of barriers) or the Wilcoxon rank-sum test (number of barriers).

Because potentially modifiable barriers related to knowledge deficits were the only barrier type significantly associated with participation in breast cancer detection practices, we further analyzed this relationship separated by individual question (Table 4). Agreeing with the statement “I don’t know where I should go if I want to get a breast exam,” was the only question showing a significant unadjusted association with lower participation in breast cancer education (p<0.001) and BSE (p = 0.021) in the cohort. The association with breast cancer education remained significant after adjusting for rural vs urban setting and age (p<0.001) while the association with BSE was no longer statistically significant after adjustment (p = 0.1) (S3 Table). After the setting and age adjustments, associations between BSE and agreement with the statements “I only need a breast exam if I have a breast problem” (p = 0.030) and “I do not need a breast exam because I feel fine” (p = 0.027) became marginally statistically significant while they were marginally not statistically significant during the unadjusted comparisons in Table 4 (p = 0.087 and p = 0.11, respectively).

**Table 4:** Knowledge deficits as barriers to breast cancer education and regular BSE.

		Any Breast Cancer Education†			Regular BSE†		
Barriers: Knowledge-deficits	No. of Responses	Yes (N = 155)	No (N = 186)	P-value‡	Yes (N = 92)	No (N = 249)	P-value‡
A breast exam is not recommended for women my age.	338	33 (21.3)	35 (19.1)	0.68	16 (17.6)	52 (21.1)	0.54
I only need a breast exam if I have breast problem.	341	71 (45.8)	86 (46.2)	>0.99	35 (38.0)	122 (49.0)	0.087
I don’t know where I should go if I want to get a breast exam.	340	59 (38.3)	113 (60.8)	**<0.001**	37 (40.2)	135 (54.4)	**0.021**
I don’t need a breast exam from a doctor because I examine my own breasts.	336	19 (12.4)	14 (7.7)	0.20	8 (8.7)	25 (10.2)	0.84
I do not need a breast exam because I feel fine.	337	18 (11.7)	28 (15.3)	0.43	8 (8.7)	38 (15.5)	0.11

BSE = breast self-exam;

^†^Values are no. (%) or mean ± SD;

^‡^Fisher’s exact test (presence of barriers) or the Wilcoxon rank-sum test (number of barriers).

## Discussion

In this study, we evaluated barriers Ugandan women without breast cancer encounter with different breast cancer detection practices as a proxy for understanding the barriers women with breast cancer who are less likely to achieve a diagnosis may encounter along the path to diagnosis. Previous studies showed multiple largely socially determined sociodemographic factors and infrastructure barriers associated with delays in diagnosis, however, we showed that knowledge deficits—a modifiable factor- was the only barrier associated with participating in breast cancer detection practices. These results suggest that program planners in SSA developing early breast cancer detection plans should first focus on strengthening population breast cancer education efforts to improve breast cancer outcomes.

Previous studies in predominantly urban Ugandan women with breast cancer who accessed treatment have shown economic barriers to access, poor social support, and other socially determined barriers are prevalent and potentially responsible for poor breast cancer outcomes in SSA. While we similarly found these barriers to be prevalent in the urban and rural Ugandan women without breast cancer, they were not significantly associated with women’s participation in breast cancer detection practices. We found that only the modifiable barrier knowledge deficits was associated with participation in breast cancer detection practices. Differences between these studies may be due in part to differences in populations (i.e. our inclusion of rural women) and, in particular, that previous studies focused on the minority of women with breast cancer who successfully accessed treatment, who may not be representative of the broader population of women without breast cancer or those with breast cancer who do not achieve a diagnosis. Another possibility is that barriers not perceived as important by women without a diagnosis become important once diagnosed with breast cancer. Regardless, our results suggest that women without breast cancer represent a distinct population to be considered during program planning for early breast cancer detection in countries where most women do not access treatment.

Knowledge deficits were significantly associated with decreased prior breast cancer education and performing BSE—both breast cancer detection practices associated with reduced patient delay. The African Breast Cancer Disparities Outcomes study[1] discovered multiple associations between tumor stage and measures of breast cancer knowledge. Specifically, less breast cancer knowledge led to continual increase in the risk for presenting for treatment at a late stage, and nearly 1 in 5 participants who presented at late stage had never heard of breast cancer. The association between breast cancer knowledge and breast cancer detection practices in the patient interval of both urban and rural women suggests that low-cost education-based interventions could be nationally efficacious.

One notable finding was that overall 51% of women reported that they did not know or were unsure of where to go for a breast examination. This was somewhat surprising because the Ugandan government has built community health centers, in catchment areas of approximately 84,000 people,[29] to provide access to basic healthcare (e.g., antibiotics for diarrheal illnesses, malaria treatment). Because of their proximity and services provided, women are typically familiar with the location of their local community health centers. Our results indicate that many women do not seem to know they can receive a breast examination at their community health center or this service is not offered at these locations and that this lack of knowledge is associated with lower participation in breast cancer detection practices. While health providers at community health centers receive formal education in performing breast examinations, they do not receive updates or mentoring and, thus, some feel uncomfortable with their skills.[30] Instead, they either refer patients with breast problems to a hospital, located far away, but staffed by a physician, or choose to locally follow women. Perhaps women in the community perceive their provider’s discomfort or that “nothing is being done” locally. These factors may contribute to the diagnostic delays associated with late stage diagnosis. These symptomatic women with undiagnosed breast cancer may remain local and/or discontinue their diagnostic work-up (i.e. lost to follow-up). The diagnostic interval represents a critical time to intervene and downstage breast cancer.

We found that urban women reported significantly more fear-based barriers than rural women, with similar economic barriers to access and poor social support. Yet, urban women were simultaneously more likely to participate in breast cancer detection practices at all intervals along the pathway to diagnosis. While there may be underlying confounding factors not elicited in this study, ranging from ethnic or religious differences to greater levels of fear-associated beliefs serving as a call to action, the prevalence of fear-based barriers in urban women without breast cancer suggest that little comfort was derived from participation in breast cancer detection practices. Instead, these findings suggest that breast cancer education could at least partially mitigate or compensate for socially determined barriers such as economic barriers to access, fear and poor social support. These findings also support the BGHI resource-stratified guidelines recommending low-cost strategies, such as strengthening breast cancer education efforts, at the basic level.

Previous studies have used or recommended education as a core component of a comprehensive breast cancer strategy.[1, 6, 9–11] Despite these efforts, women continue to present predominantly with late stage breast cancer in Uganda and other countries in SSA.[31] These studies suggest that current efforts are inadequate to address barriers thwarting early breast cancer detection. The ASK study was designed to provide more accurate and specific data to help design improved breast health messages for Ugandan women. Future efforts by UWOCASO will translate these data into more effective breast cancer education to improve population awareness. Simultaneously, our findings suggest that education efforts should extend beyond population awareness and include providers at community health centers, so women can receive CBE and accurate breast information near their homes.

Socially determined factors, such as economic barriers to accessing care, were common in this study and may be difficult to address.[1, 7, 9] Constraints in time, lost potential wages or productivity to seek medical evaluations for preventative care, and the difficulty in accessing medical care, particularly imaging and pathology services, all create a milieu in which the expense of receiving healthcare extends beyond the “cost” of the visit. Shifting from a centralized system to a decentralized one where part of the diagnostic work-up is offered at community health centers may mitigate this ubiquitous barrier. Alternatively, engaging village health teams (local volunteer women educated in health matters and familiar with the health system) to act as patient navigators may ensure more women complete their work-ups at higher level health centers offering these services. Such interventions may reduce the diagnostic interval and help downstage breast cancer at treatment. Matovu, *et al* previously showed how providing free diagnostic ultrasound locally to women with positive breast examinations reduced the proportion of women with breast cancer diagnosed at a late stage compared to historical averages.[32] This approach may be too expensive to offer every day at every community health center; however, periodic offering with newer portable ultrasound technology, coupled with evaluation of other medical conditions (e.g., obstetrics), may provide a resource-appropriate solution to a growing health crisis in sub-Saharan Africa.[9] Further work is indicated to delineate referral patterns of providers at community health centers and costs and benefits associated with each solution.

This study was limited by its convenience-based sampling and potential generalizability to our study population (women with undiagnosed breast cancer). At least 76% of our surveyed women were below the international poverty line. While Uganda has reduced the population living below the poverty line from 54% (2006) to 36% (2013),[33] 70% of Ugandans remain poor or highly vulnerable to poverty.[34] Poor and vulnerable women are more likely to experience diagnostic delays and not present for treatment. More affluent women do not have the same barriers to accessing breast cancer detection and diagnosis as vulnerable women. Prior studies were confounded by sampling bias by including predominantly urban women with access to a tertiary care center; our inclusion of rural population is closer to the true population distribution of Uganda (80% rural). In addition, the low numbers of participants with a history of CBE, breast ultrasound, or biopsy, particularly among rural women, limited our ability to draw reliable conclusions associated with barriers to accessing breast cancer detection practices related to the diagnostic interval. However, the rates of CBE in urban women (36%) is similar to a prior study in Ugandan women seeking care at a major tertiary hospital.[28] Therefore, while our sample may not be generalizable to the entire Ugandan population, it provides a reasonable estimate of our study population (women with undiagnosed breast cancer), particularly the most vulnerable women most likely to experience diagnostic delays and poor outcomes.

While many barriers to successful progression to diagnosis are not easily addressed in Uganda, improving women’s knowledge is a critical, realistic, achievable goal in Uganda and could mitigate the effects of prevalent socially determined factors associated with delayed diagnosis. Addressing this low-hanging fruit could dramatically reduce morbidity and mortality associated with late-stage breast cancer in resource-limited countries.

## Supporting information

S1 Table Comparison of type and number of barriers between urban and rural women. BSE = breast self-exam; CBE = clinical breast exam; US = breast ultrasound; †Values are no. (%) or mean ± SD; ‡Fisher’s exact test (presence of barriers) or the Wilcoxon rank-sum test (number of barriers).
(DOCX)

S2 Table Adjusted associations between patient-related barriers and participation in breast cancer detection practices. BSE = breast self-exam; CBE = clinical breast exam; US = breast ultrasound; PD = probability difference (outcome with barrier minus outcome without barrier); *Based on binomial regression models with breast cancer detection practice as the outcome variable; covariates include an individual barrier type (present vs. absent), age, and urban vs. rural.
(DOCX)

S3 Table Adjusted associations between knowledge deficits as barriers and participation in breast cancer detection practices. BSE = breast self-exam; PD = probability difference (outcome with barrier minus outcome without barrier); *Based on binomial regression models with breast cancer detection practice as the outcome variable; covariates include an individual barrier type (present vs. absent), age, and urban vs. rural.
(DOCX)
