# Supplementary material for: Modifiable patient-related barriers and their association with breast cancer detection practices among Ugandan women without a diagnosis of breast cancer
Source: PLoS One. 2019 Jun 20;14(6):e0217938. doi: 10.1371/journal.pone.0217938 (PMC6586444; doi:10.1371/journal.pone.0217938)
Supplement: S1 Table — BSE = breast self-exam; CBE = clinical breast exam; US = breast ultrasound; †Values are no. (%) or mean ± SD; ‡Fisher’s exact test (presence of barriers) or the Wilcoxon rank-sum test (number of barriers). (DOCX) [file pone.0217938.s001.docx]

**S1 Table. Comparison of Type and Number of Barriers Between Urban and Rural Women.**

|  | **Location**† | |  |
| --- | --- | --- | --- |
| **Variable** | **Urban**  **(N=83)** | **Rural**  **(N=258)** | **P-value‡** |
| Barriers |  |  |  |
| Economic | 55 (66.3) | 178 (69.0) | 0.68 |
| Social support | 20 (24.1) | 62 (24.0) | >0.99 |
| Knowledge | 62 (74.7) | 208 (80.6) | 0.28 |
| Fear/psychological | 43 (51.8) | 84 (32.6) | **0.003** |
|  |  |  |  |
| Number of barrier types | 2.2 ± 1.2 | 2.1 ± 1.1 | 0.44 |

BSE = breast self-exam; CBE = clinical breast exam; US = breast ultrasound;

†Values are no. (%) or mean ± SD;

‡Fisher’s exact test (presence of barriers) or the Wilcoxon rank-sum test (number of barriers).
