# Supplementary material for: Modifiable patient-related barriers and their association with breast cancer detection practices among Ugandan women without a diagnosis of breast cancer
Source: PLoS One. 2019 Jun 20;14(6):e0217938. doi: 10.1371/journal.pone.0217938 (PMC6586444; doi:10.1371/journal.pone.0217938)
Supplement: S2 Table — BSE = breast self-exam; CBE = clinical breast exam; US = breast ultrasound; PD = probability difference (outcome with barrier minus outcome without barrier); *Based on binomial regression models with breast cancer detection practice as the outcome variable; covariates include an individual barrier type (present vs. absent), age, and urban vs. rural. (DOCX) [file pone.0217938.s002.docx]

**S2 Table. Adjusted Associations Between Patient-Related Barriers and Participation in Breast Cancer Detection Practices.**

|  | **Breast**  **Cancer Education*** | | |  | **Regular BSE*** | | |  | **Recent CBE or Prior**  **Breast US or Biopsy*** | | |
| --- | --- | --- | --- | --- | --- | --- | --- | --- | --- | --- | --- |
| **Barriers** | **PD** | **(95% CI)** | **P-value** |  | **PD** | **(95% CI)** | **P-value** |  | **PD** | **(95% CI)** | **P-value‡** |
| Economic barriers | -0.09 | (-0.20, 0.02) | 0.11 |  | -0.05 | (-0.14, 0.05) | 0.36 |  | 0.00 | (-0.09, 0.08) | 0.94 |
| Poor social support | 0.01 | (-0.11, 0.14) | 0.85 |  | -0.07 | (-0.17, 0.04) | 0.20 |  | 0.06 | (-0.04, 0.17) | 0.22 |
| Knowledge deficits | -0.17 | (-0.29, -0.05) | **0.005** |  | -0.18 | (-0.30, -0.05) | **0.005** |  | -0.08 | (-0.19, 0.02) | 0.12 |
| Fear | 0.00 | (-0.10, 0.11) | 0.94 |  | -0.02 | (-0.12, 0.08) | 0.71 |  | 0.02 | (-0.07, 0.11) | 0.71 |

BSE = breast self-exam; CBE = clinical breast exam; US = breast ultrasound; PD = probability difference (outcome with barrier minus outcome without barrier);

*Based on binomial regression models with breast cancer detection practice as the outcome variable; covariates include an individual barrier type (present vs. absent), age, and urban vs. rural.
