# Supplementary material for: Modifiable patient-related barriers and their association with breast cancer detection practices among Ugandan women without a diagnosis of breast cancer
Source: PLoS One. 2019 Jun 20;14(6):e0217938. doi: 10.1371/journal.pone.0217938 (PMC6586444; doi:10.1371/journal.pone.0217938)
Supplement: S3 Table — BSE = breast self-exam; PD = probability difference (outcome with barrier minus outcome without barrier); *Based on binomial regression models with breast cancer detection practice as the outcome variable; covariates include an individual barrier type (present vs. absent), age, and urban vs. rural. (DOCX) [file pone.0217938.s003.docx]

**S3 Table. Adjusted Associations Between Knowledge Deficits as Barriers and Participation in Breast Cancer Detection Practices.**

|  |  |  | **Any Breast**  **Cancer Education*** | |  |  | **Regular BSE*** | |  |
| --- | --- | --- | --- | --- | --- | --- | --- | --- | --- |
| **Barriers: Knowledge-deficits** | **No. of**  **Responses** |  | **PD** | **(95% CI)** | **P-value** |  | **PD** | **(95% CI)** | **P-value** |
| A breast exam is not recommended for women my age. | 338 |  | 0.00 | (-0.14, 0.13) | 0.97 |  | -0.08 | (-0.19, 0.03) | 0.17 |
| I only need a breast exam if I have breast problem. | 341 |  | -0.02 | (-0.12, 0.08) | 0.72 |  | -0.10 | (-0.19, -0.01) | **0.030** |
| I don’t know where I should go if I want to get a breast exam. | 340 |  | -0.19 | (-0.29, -0.08) | **<0.001** |  | -0.08 | (-0.17, 0.02) | 0.10 |
| I don’t need a breast exam from a doctor because I examine my own breasts. | 336 |  | 0.11 | (-0.08, 0.31) | 0.25 |  | -0.05 | (-0.20, 0.10) | 0.50 |
| I do not need a breast exam because I feel fine. | 337 |  | -0.09 | (-0.25, 0.06) | 0.24 |  | -0.13 | (-0.24, -0.01) | **0.027** |

BSE = breast self-exam; PD = probability difference (outcome with barrier minus outcome without barrier);

*Based on binomial regression models with breast cancer detection practice as the outcome variable; covariates include an individual barrier type (present vs. absent), age, and urban vs. rural.
